# Quantitative Property-Property Relationship for Screening-Level Prediction of Intrinsic Clearance of Volatile Organic Chemicals in Rats and Its Integration within PBPK Models to Predict Inhalation Pharmacokinetics in Humans

**DOI:** 10.1155/2012/286079

**Published:** 2012-05-22

**Authors:** Thomas Peyret, Kannan Krishnan

**Affiliations:** Département de Santé Environnementale et Santé au Travail, Université de Montréal, C.P. 6128, Succursale Centre-Ville, Montréal, QC, Canada H3C 3J7

## Abstract

The objectives of this study were (i) to develop a screening-level Quantitative property-property relationship (QPPR) for intrinsic clearance (CL_int_) obtained from *in vivo* animal studies and (ii) to incorporate it with human physiology in a PBPK model for predicting the inhalation pharmacokinetics of VOCs. CL_int_, calculated as the ratio of the *in vivo V*_max_ (*μ*mol/h/kg bw rat) to the *K*_*m*_ (*μ*M), was obtained for 26 VOCs from the literature. The QPPR model resulting from stepwise linear regression analysis passed the validation step (*R* ^2^ = 0.8; leave-one-out cross-validation *Q* ^2^ = 0.75) for CL_int_ normalized to the phospholipid (PL) affinity of the VOCs. The QPPR facilitated the calculation of CL_int_ (L PL/h/kg bw rat) from the input data on log *P*_ow_, log blood: water PC and ionization potential. The predictions of the QPPR as lower and upper bounds of the 95% mean confidence intervals (LMCI and UMCI, resp.) were then integrated within a human PBPK model. The ratio of the maximum (using LMCI for CL_int_) to minimum (using UMCI for CL_int_) AUC predicted by the QPPR-PBPK model was 1.36 ± 0.4 and ranged from 1.06 (1,1-dichloroethylene) to 2.8 (isoprene). Overall, the integrated QPPR-PBPK modeling method developed in this study is a pragmatic way of characterizing the impact of the lack of knowledge of CL_int_ in predicting human pharmacokinetics of VOCs, as well as the impact of prediction uncertainty of CL_int_ on human pharmacokinetics of VOCs.

## 1. Introduction

The evolving scientific and regulatory activities in Europe and North America emphasize the need for the development of tools that refine, replace, or reduce the use of animals and human volunteers in pharmacokinetic and toxicity tests [1–3]. The ability to base the toxic responses on the target tissue dose or internal concentration of the toxic moiety of the chemicals is key to the predictive tools reflective of the current state of science. Therefore, physiologically based pharmacokinetic (PBPK) models that are capable of providing *a priori* prediction of the time course of chemicals in blood and tissues is of tremendous interest [4]. PBPK models are mechanistically based mathematical descriptions of the absorption, distribution, metabolism, and excretion of chemicals or pharmaceutical compounds. In PBPK models, the organism is represented as a set of several tissue compartments interconnected by blood flows. In these models, the internal dose measures (e.g., blood or tissue concentrations, amount metabolized) of a chemical are described on the basis of mass-balance differential equations requiring species-specific properties (e.g., alveolar ventilation rate, cardiac output, regional blood flows, and tissue volumes) and chemical-specific input parameters (e.g., partition coefficients and metabolic constants). Although the species-specific values of several physiological parameters are available in the literature [4–6], the partition coefficients (PCs) and metabolic constants need to be determined experimentally or calculated by using animal-replacement methods for each chemical individually [7]. The values of tissue : blood or tissue : plasma partition coefficients essential for developing PBPK models have been estimated for a wide range of chemicals and chemical classes, including drugs, with the use of tissue composition-based algorithms or QSAR methods (e.g., [8–19]). 

Regarding the metabolism parameters (i.e., hepatic clearance, intrinsic clearance, *V*_max⁡_, *K*_*m*_, *K*_cat_, free energy of binding, energy of activation, or activation enthalpy), some studies have developed 2-D and 3-D QSARs but with a specific focus on either a single isozyme, a single reaction or a single class of substances [8, 20–38]. None of these past efforts succeeded in predicting both *V*_max⁡_ and *K*_*m*_ (or *CL*_int⁡_) of environmental chemicals for direct incorporation within animal or human PBPK models. Alternatively, few studies utilized the group contribution method of Gao [39–43], to predict metabolic rates for PBPK models. In this method, the chemical is decomposed into different structural fragments or groups, the contributions of which are obtained by regression analysis [39]. Accordingly, these publications demonstrated the feasibility of developing structure-property relationships for the metabolism rates. The group contribution method was successfully used to develop quantitative structure-property relationships (QSPRs) for the tissue : air partition coefficients as well as intrinsic (*CL*_int⁡_) and hepatic clearance (*CL*_*h*_ for a group of low-molecular-weight volatile organic chemicals (VOCs) in rats [41, 42]. These QSPR models, in turn, were incorporated within PBPK models to predict reasonably well the blood kinetics of inhaled VOCs in rats. As these QSPRs are species specific, they could not be used to conduct interspecies extrapolations. To overcome this limitation, Béliveau et al. [40] developed biologically based algorithms for PCs and *CL*_*h*_ to conduct rat to human extrapolations of the inhalation toxicokinetics of VOCs. In this study, QSPRs based on the group contribution method were developed for the chemical-specific input parameters of the biological algorithms for PCs (i.e., oil : air, water : air,and blood protein : air) and *CL*_int⁡_ (intrinsic clearance normalized for cytochrome P450 2E1 content). More recently, QSPRs were developed for the metabolic constants *V*_max⁡_ (maximum velocity of reaction) and *K*_*m*_ (Michaelis constant) [43] and were further incorporated within a rat PBPK model to predict the toxicokinetics of mixtures of VOCs. Despite the successful use of the group contribution method in QSPR modeling of metabolism rates, their principal limitation relates to the fact that the chemical space they cover is extremely limited (low-molecular-weight VOCs containing one or more of the following fragments: CH_3_, CH_2_, CH, C, C=C, H, Br, Cl, F, benzene ring, and H on benzene ring). More experimental data on diverse chemicals would be needed to determine the contributions of other molecular fragments, as has been done with *P*_ow_ (e.g., estimation of the contribution of 130 fragments (i.e., groups) required 1200 measurements of *P*_ow_) [44]. To extend the currently available QSPR for *CL*_int⁡_ to cover more diverse fragments and at the same time respect a reasonable ratio of the number of parameters to the number of observations, extensive experimental data would be required. 

Since the critical limitation in the construction of PBPK models for new substances continues to be the metabolism rate, a pragmatic approach—particularly for inhaled VOCs—is to evaluate the maximum and minimum possible blood concentration profiles in exposed individuals. Thus, using a hepatic extraction ratio (*E*) of 0 and 1 in the PBPK models, Poulin and Krishnan [45] obtained simulations of the physiological limits (i.e., maximal and minimal blood concentration profiles) for inhaled VOCs in humans. Assuming the conceptual PBPK model and the values of its physiological parameters are reliable, the real answer, that is, the actual concentrations and kinetic curve, would be somewhere in between the theoretical limits simulated with these PBPK models [45]. The uncertainty associated with these theoretical bounds can be reduced by developing better estimates of the metabolism constants. This could be done, at a practical level, by developing *in silico* tools that provide a range of plausible values, in lieu of a single accurate point estimate. Such a tool might be of use for the toxicokinetic screening of substances, until the time when the chemical-specific measurements are obtained *in vivo*, *in vitro,* or with a highly precise mechanistic *in silico* method. 

Since human exposures to environmental contaminants in most cases do not attain levels that approach or exceed saturation, it is not crucial to predict *V*_max⁡_ and *K*_*m*_ separately, particularly for simulating kinetics in humans exposed to low atmospheric concentrations of VOCs. Therefore, the availability of *in silico* approaches based on easily available parameters to predict plausible range of *CL*_int⁡_ would be desirable as a screening-level tool. The objective of this study was therefore to develop a quantitative property-property relationship (QPPR) model of animal data to generate initial estimates (or bounds) of intrinsic clearance of VOCs, for eventual incorporation within a human PBPK model to simulate blood concentration profiles associated with inhalation exposures. In this regard, we focused on evaluating the impact of the uncertainty associated with QPPR predictions of *CL*_int⁡_ on the blood kinetics of VOCs in humans, relative to that of the uncertainty associated with the total lack of knowledge of the metabolic rate in humans. Furthermore, the reliability of applying the QPPR to predict the area under the blood concentration versus time curve (AUC) of parent chemicals was evaluated, as a function of the sensitivity of the metabolism parameter in the PBPK model and the prediction uncertainty of QPPR model.

## 2. Methods

A QPPR model for *CL*_int⁡_ was developed using a calibration set of 26 VOCs. The QPPR predictions were then compared with experimental data for several VOCs and the pharmacokinetics in humans were simulated using integrated QPPR-PBPK models for these 26 VOCs. The predictions of QPPR were evaluated further with an external data set of *CL*_int⁡_ for 11 VOCs.

### 2.1. QPPR Modeling for Intrinsic Clearance

#### 2.1.1. Chemicals and Data Sources

The development of a global QPPR model for metabolism was initially undertaken using experimental data on the *in vivo* intrinsic clearance of 26 VOCs in rats, collated and evaluated in previous studies by Béliveau et al. [40, 41] (1,1,1,2-tetrachloroethane; 1,1,2,2-tetrachloroethane; 1,1,2-trichloroethane; 1,1-dichloroethane; 1,1-dichloroethylene; 1,2-dichloroethane; benzene; bromochloromethane; bromodichloromethane; carbon tetrachloride; chloroethane; chloroform; *cis*-1,2-dichloroethylene; dibromomethane; dichloromethane; ethylbenzene; hexachloroethane; isoprene; methyl chloride; *m*-xylene; *n*-hexane; pentachloroethane; styrene; toluene; trichloroethylene; vinyl chloride) [24, 46–53].

Subsequently, the resulting QPPR model was evaluated with experimental *in vivo* data on *CL*_int⁡_ for 11 additional VOCs in rats (1,1,1-trichloroethane; 1,2,4-trimethylbenzene; bromoform; dibromochloromethane; furan; halothane; *o*-xylene; *trans*-1,2-dichloroethylene; tetrachloroethylene; propylene; ethylene) [46, 48, 54–61]. These 11 chemicals outside the calibration set were also lipophilic, low-molecular-weight VOCs and likely substrates of cytochrome P450 2E1 [32, 62]. Moreover except for halothane and 1,2,4-trimethylbenzene, the chemicals of the evaluation dataset possess values of *P*_ow_, ionization potential, and blood : water PC within the range of values for the chemicals in the QPPR calibration set.

#### 2.1.2. Modeling Endpoint

For QPPR modeling, *CL*_int⁡_ (expressed in units of L blood, *CL*_intblood_, or L phospholipids, *CL*_intPL_) was used as the endpoint. Initially, *CL*_intblood_ (L  blood/h/kg^0.75^) for all the studied chemicals was computed as allometrically scaled *V*_max⁡_ (*μ*mol/h/kg^0.75^)/*K*_*m*_ (*μ*mol/L  blood). Since CYPs are located in the endoplasmic reticulum embedded in the phospholipidic bilayer [63], the *CL*_intPL_  values reflecting chemical affinity for the phospholipids (PL) were subsequently computed. The values of *CL*_intPL_ (L  phospholipid/h/kg^0.75^) were obtained by dividing *V*_max⁡_ (*μ*mol/h/kg^0.75^) with *K*_*m*_ expressed as *μ*mol/L  PL. The *K*_*m*_ values in *μ*M of PL were obtained by multiplying the values of *K*_*m*_ expressed as *μ*mol/L  blood with the chemical-specific phospholipid : blood partition coefficients (*P*_plb_) calculated as follows:

(1) $$P_{\text{plb}}=\frac{0.3\cdot P_{\text{oa}}+0.7\cdot P_{\text{wa}}}{P_{\text{ba}}},$$

where *P*_oa_ is the *n*-octanol : air PC, *P*_wa_ the water : air PC, and *P*_ba_ the blood : air PC.

The above equation computes *P*_plb_ as the ratio of phospholipid : air to blood : air PCs of the VOCs, based on Poulin and Krishnan [10, 12].

#### 2.1.3. Input Parameters for Transforming the Endpoint

The input parameters required for converting the *CL*_int⁡_  obtained from the literature were *P*_oa_, *P*_wa_, and *P*_ba_.

**(1) *P*_oa_ and *P*_wa_**

The *n*-octanol : air PC (*P*_oa_), was calculated as the product of the *n*-octanol : water PC (*P*_ow_) and *P*_wa_ (inverse of Henry's law constant at 37.5°C). The values of  *P*_ow_ and *P*_wa_ were predicted using U.S. EPA's freeware EPISUITE (http://www.epa.gov/opptintr/exposure/pubs/episuite.htm).

**(2) *P*_ba_**

Experimental values were used for rat blood : air [54, 56, 59, 64–68]. The calculated values of *P*_plb_ for the chemicals used for the development and for the evaluation of the QPPR are reported in Table 1.

#### 2.1.4. Variable Selection

*A priori* list of variables was developed on the basis on mechanistic considerations. The rate and affinity for P450-mediated metabolism would appear to be related to the size, shape, charge, and energy of the substrate; therefore variables that reflect these properties were chosen for the QPPR analysis [21, 23, 27, 28, 32, 69–71]. The descriptors of the size and shape of the molecule were the molecular length, width, depth, volume, surface, and the Kappa 2 index [72], as well as two descriptors used in the work of Lewis et al. [23], namely, the ratio of the molecular length to the molecular width (L/W) and the ratio of the area of the molecule (i.e., length times width) to the square of the depth (*a*/*d* ^2^). The dipole moment and ionization potential (IP) were used as measure of the charge disposition and the energy in the molecule, respectively. The values of all the previously cited descriptors were calculated using commercially available software (Molecular Modeling Pro, Chem SW, Fairfield, CA). Before calculating the molecular descriptors with Molecular Modeling Pro, the 3D molecules were drawn and minimized using the full MM2 (molecular mechanics program) method provided in the software. The dipole moment and the ionization potential were calculated using MOPAC/PM3 program, included in Molecular Modeling Pro. 

Hydrophobic descriptors such as log *P*_ow  _(log of the *n*-octanol : water PC) that reflect hydrogen bonding and *π*-*π* stacking have already been correlated to the values of metabolic constants [69–71]. In this study, the following physicochemical parameters were chosen to describe the relative solubility and partitioning into diverse biological media: log *P*_ow_, log phospholipid : water PC (log *P*_plw_); log blood : water PC (log *P*_bw_), and log water : air PC (log *P*_wa_). The blood : water and phospholipid : water PCs were obtained by dividing the blood : air and phospholipid : air PCs values by the water : air PC values. The values of *P*_ow_, *P*_wa_, blood : air, and phospholipid : air PCs were obtained as described for the calculation of *P*_plb_ (1).

#### 2.1.5. Statistical Analysis

Multilinear regression analysis approach was chosen for the QPPR analysis of *CL*_int⁡_ because linear regression models are simple, transparent, and easy to reproduce [73]. The regression analysis was performed using SPSS v16 for Windows (SPSS Inc., Chicago, IL). Stepwise regression analysis was performed to select the QPPRs based on the most statistically significant independent variable(s) from an *a priori* list (see Section 2.1.4). The coefficient of determination *R* ^2^, the adjusted *R* ^2^ (*R*_adj_^2^; adjusted for number of variables) [73], the standard error of the estimate *s*, and the value and significance of the *F*  statistic were calculated. The normality of the residuals was checked visually on normal probability plots of the standardized residuals (i.e., expected normal cumulative probability versus observed cumulative probability). Leave-one-out cross-validation was conducted and the results were expressed in terms of *Q* ^2^, a measure of precision error of the model. The *Q* ^2^ was computed as follows [74]:

(2) $$Q^{2}=1-\frac{\text{PRESS}}{\text{SSY}},$$

where PRESS is that predicted residual sum of squares and SSY the sum of squares of the response values. The statistical significance (*p* < 0.05) of the regression coefficients was estimated by a *t* statistic test. Multicollinearity refers to the occurrence of correlation between two independent variables in the multiple linear regression model. Multicollinearity of the variables in the model was assessed by calculating the variance inflation factor (VIF) for all independent variables [75]. The value of VIF was calculated as follows [75]:

(3) $${\text{VIF}}_{i}=\frac{1}{1-R_{i}^{2}},$$

where VIF_*i*_ is the variance inflation factor of the independent variable *i* in the multilinear regression model and *R*_*i*_^2^ the coefficient of determination of the regression between the independent variable *i* and the other independent variables in the multilinear regression model.

For each model, the application domain was documented by reporting the ranges of values of the descriptors, the modeled response, and the endpoint.

A QPPR model was considered adequate when: the values of *R* ^2^ and *R*_adj_^2^ were ≥0.6 [73], the value of *Q* ^2^ was ≥0.6 [76], and the independent variables were not highly correlated (i.e., VIF < 4) [75].

The predictions of the QPPR model were obtained in terms of lower and upper bounds of the 95% mean confidence intervals (LMCI and UMCI, resp.) in order to represent the uncertainty associated with the mean predicted value. The LMCI and UMCI for the 11 VOCs, not in the QSPR calibration dataset, were obtained by adding them in the SPSS file containing the data used for the QPPR, along with the values of their independent variables only.

### 2.2. Translation of QPPR Predicted Intrinsic Clearance Values to *In Vivo* Metabolism Rate and Integration within Human PBPK Models

In the PBPK model, the value of intrinsic clearance was calculated as the product of the QPPR value of *CL*_intPL_ (L  of  PL/h/kg^0.75^) and the phospholipid : blood PC (values of *P*_plb_ in Table 1). The intrinsic clearance (L  blood/h/kg^0.75^) was used within the human PBPK models to compute the hepatic clearance.

The rate of metabolism was calculated on the basis of hepatic clearance (i.e., hepatic clearance times the arterial concentration) [4, 40, 41, 45]. For chloroethane, dichloromethane, vinyl chloride, and dibromomethane a first-order constant (1, 2, 1, and 0.7 h^−1^, resp.) was included in the calculation of the hepatic clearance, *CL*_*h*_  (L/h) [41]:

(4) $${CL}_{h}=Q_{L}\cdot E,$$

where *E* = (*CL*_int⁡_ + *K*_*f*_ · *V*_*L*_)/((*CL*_int⁡_ + *K*_*f*_ · *V*_*L*_) + *Q*_*L*_), Q_L_ is the blood flow through the liver (L/h), *CL*_int⁡_ the intrinsic clearance (L blood/h), *K*_*f*_ the first order metabolic constant (h^−1^), and V_L_ the  liver volume (L). 

### 2.3. PBPK Modeling

The QPPR values of *CL*_int⁡_ were included in a human PBPK model for inhaled VOCs [50]. Briefly, the PBPK model consisted in four tissue compartments (i.e., liver, fat, richly, and poorly perfused tissues) and a gas exchange lung, which were interconnected by blood flows. The distribution of VOCs into tissue compartments was described as perfusion limited, and the metabolism was limited to liver.

To evaluate the impact of uncertainty on the metabolic rate, for all the chemicals, PBPK simulations were also conducted by setting the value of *E* to 0.999 (*E*_max⁡_) and then to 0.001 (*E*_min⁡_), respectively.

The human physiological parameters of the PBPK model (i.e., body weight = 70 kg; cardiac output = 18 L/h/kg^0.74^; alveolar ventilation = 18 L/h/kg^0.74^; tissue compartment volumes, fraction of body weight:liver = 0.026; richly perfused tissues = 0.05; poorly perfused tissues = 0.62; fat = 0.19; perfusion of the tissue compartments, fraction of cardiac output:liver = 0.26; richly perfused tissues = 0.44; poorly perfused tissues = 0.25; fat = 0.05) were obtained from Tardif et al. [67]. Table 1 presents the value of the partition coefficients used in the PBPK model (i.e., blood : air, tissue : blood, and phospholipid : blood PCs). The phospholipid : blood PC was calculated using (1), whereas the blood : air PC and tissue : blood PCs were gathered from the literature [48, 50, 52, 54, 56–59, 61, 66, 67, 77–80].

The PBPK model (differential and algebraic mass-balance equations, physiological parameters, QSPR equations for metabolic constants, and PCs) was written in ACSL (acslX, version 2.5, Aegis Technologies Group, Inc, Huntsville, AL). The model code is included in the supplementary data available online at doi:10.1155/2012/286079. To compare the impact of different (uncertain) scenarios of rate of metabolism on the pharmacokinetics in human, simulations were carried out by setting (i) the value of *CL*_int⁡_ equal to the lower and upper bound of the QPPR predicted mean 95% confidence interval, or (ii) the liver extraction ratio to 0.001 (no metabolism) and 0.999 (maximum extraction). The 24 h venous blood kinetics corresponding to the four scenarios of metabolism were simulated for an 8 h exposure to 1 ppm of each VOC. The 24 h area under the curve (AUC_24_) of the venous blood kinetics was also calculated to compare the four scenarios of metabolism simulated with PBPK models. Additionally, the venous blood kinetics of *m*-xylene, toluene, ethylbenzene, dichloromethane, styrene, 1,2,4-trimethylbenzene, and 1,1,1-trichloroethane were compared to experimental data [61, 67, 81–83].

### 2.4. Analysis of Applicability of the *CL*_int⁡_ QPPR to PBPK Modeling

**Figure 1:**
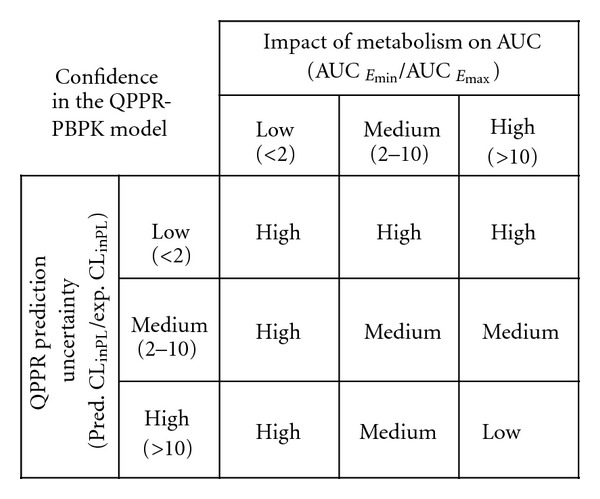
Evaluation of the confidence in applying the QPPR for *CL*_intPL_ in a PBPK model using a sensitivity/uncertainty approach.

The applicability of the QPPR model was evaluated on the basis of the level of uncertainty in the QPPR estimate and the impact (sensitivity) of metabolism on the AUC_24_.  Figure 1 illustrates the role of uncertainty and sensitivity in the reliability of the QPPR-PBPK modeling framework, based on reference [84]. The sensitivity of the metabolism to the AUC was estimated by the ratio of the AUC_24_ obtained with no metabolism (*E*_min⁡_) to that obtained with the maximum theoretical metabolism (*E*_max⁡_). The sensitivity of AUC_24_ to metabolism was considered to be low, medium, or high if the ratio (AUC_*E*_min⁡__/AUC_*E*_max⁡__) was within a factor of 2, within an order of magnitude, or greater. The uncertainty in the QPPR prediction was evaluated by comparing it to the experimental data. The prediction uncertainty was considered to be low, medium or high if the prediction was within a factor of two, within an order of magnitude and above 10-fold of the experimental data, respectively. 

This approach was applied to evaluate the reliability of applying the QPPR within the PBPK model for two situations: (i) for the calibration set of chemicals, for which the uncertainty of the QPPR was evaluated by comparing the predictions of *CL*_intPL_ with the experimentally derived *CL*_intPL_ values and (ii) for chemicals in the evaluation dataset, for which the uncertainty in the QPPR prediction was considered to be “high”, to replicate the “data poor” situations with new or tested chemicals with unknown experimental *CL*_int⁡_ values.

## 3. Results

### 3.1. QPPR Development

The initial effort to develop a QPPR model for metabolism rate (expressed as *CL*_intblood_, in units of L blood/hr), based on a stepwise analysis of its relationship to various molecular descriptors and physicochemical properties, was not successful (not shown). Same analysis, repeated for *CL*_int⁡_ expressed in units of L PL/h (*CL*_intPL_), yielded a QPPR that consisted of log *P*_plw_, log *P*_bw_, and IP (ionization potential, eV) as input parameters. This model satisfied the criteria for an acceptable model in terms of coefficient of determination (*R* ^2^ = 0.802; *R*_adj_^2^ = 0.775), leave-one-out cross validation (*Q* ^2^ = 0.755), and multicollinearity (VIFs: log *P*_plw_ = 2.42; log *P*_bw_ = 2.38; IP = 1.04). The values of the regression coefficients were significant (*P* value < 0.001 for the constant, log *P*_plw_  and log *P*_bw_, and 0.007 for IP).

However, as the value of log *P*_ow_ can be obtained more readily than log *P*_plw_, the regression analysis was repeated by using log *P*_ow_, log *P*_bw_, and calculated IP, and it yielded the following QPPR:

(5) $$\begin{array}{cc} \log\;{CL}_{\text{intPL}} & =5.63\left(\pm 1.187\right)-1.287\left(\pm 0.149\right)\cdot \log\;P_{\text{ow}}\\ & \;+1.08\left(\pm 0.233\right)\cdot \log\;P_{\text{bw}}\\ & \;-0.328\left(\pm 0.111\right)\cdot \text{IP}. \end{array}$$

This QPPR model satisfied the criteria for an acceptable model in terms of coefficient of determination (*R* ^2^ = 0.796; *R*_adj_^2^ = 0.768), leave-one-out cross validation (*Q* ^2^ = 0.748), and multicollinearity (VIFs: log⁡*P*_ow_ = 2.42; log⁡ *P*_bw_ = 2.38; IP = 1.04). The application domain of the model can be described with [min; max] as follows: log⁡ *P*_ow_ = [1.09; 4.03]; log⁡*P*_bw_  [0.16; 2.49]; calculated ionization potential [9.13;11.28]. 

**Figure 2:**
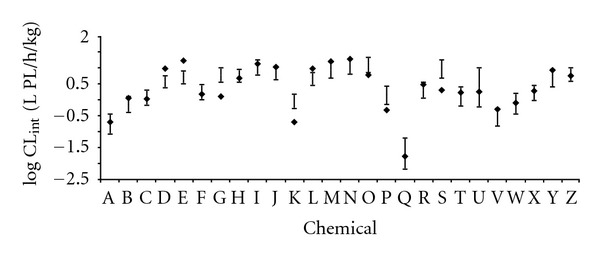
Experimental and predicted values of log *CL*_int⁡_ for 26 VOCs. The horizontal bars represent the QPPR predicted LMCI and UMCI, the symbols represent the experimental data. A: 1,1,1,2-tetrachloroethane; B: 1,1,2,2-tetrachloroethane; C: 1,1,2-trichloroethane; D: 1,1-dichloroethane; E: 1,1-dichloroethylene; F: 1,2-dichloroethane; G: 1,2-dichloroethylene (*cis*-); H: benzene; I: bromochloromethane; J: bromodichloromethane; K: carbon tetrachloride; L: chloroethane; M: chloroform; N: dibromomethane; O: dichloromethane; P: ethylbenzene; Q: hexachloroethane; R: isoprene; S: methyl chloride; T: *m*-xylene; U: *n*-hexane; V: pentachloroethane; W: styrene; X: toluene; Y: trichloroethylene; Z: vinyl chloride.

The QPPR (5) was subsequently applied to calculate the *CL*_intPL_ of the VOCs in the calibration set.  Table 2 presents the values of the input parameters, along with the experimental data for the 26 VOCs used in QPPR development.  Figure 2 illustrates the comparison of the predicted values of *CL*_intPL_ (LMCI and UMCI) and the experimental data. The uncertainty in the predicted log *CL*_intPL_ can be characterized by the difference between the UMCI and the LMCI; this value ranged from 0.37 (1,1-dichloroethane) to 1.23 (*n*-hexane) with a mean of 0.54 and a standard deviation of 0.18. The nearest confidence bounds of the predicted log *CL*_intPL_ were higher than 5-fold of the experimental value (exp.) for three substances (*cis*-1,2-dichloroethylene, LMCI = 0.55 versus exp⁡. = 0.09; styrene, LMCI = −0.45 versus exp⁡. = −0.09; and 1,1,2-trichloroethane, UMCI = 0.46 versus exp⁡. = 0.02). The impact of the imprecision of these QPPR predictions of the metabolic constants on the pharmacokinetics in humans was then evaluated by PBPK modeling.

Figure 3 presents the predictions of the 24 h blood pharmacokinetics following 8 h exposure to 1 ppm of each of the 26 VOCs used in the QPPR analysis. The bold lines represent the simulations obtained using 0 and 1 as the hepatic extraction ratio, whereas the grey area encompassed by thin lines represents the simulation obtained using LMCI and UMCI of predicted *CL*_int⁡_ in PBPK models. Overall, the envelope of the concentrations predicted using the QPPR predictions reduced the region of uncertainty associated with the complete lack of knowledge of hepatic extraction ratio in humans (i.e., ranging from 0 to 1).

The average ratio (± standard deviation) of the PBPK model simulated values of the end-of-exposure blood concentrations (i.e., *C*_max⁡_) obtained with *E*_min⁡_ and *E*_max⁡_ was 4.19 ± 1.81. The lowest and highest ratios, based on the theoretical bounds of hepatic extraction (i.e., *E*_min⁡_ and *E*_max⁡_), were observed for isoprene (1.63) and 1,1,2,2-tetrachloroethane (8.05), respectively. However, the average ratio (± standard deviation) of the PBPK model simulated values of the end-of-exposure blood concentrations, based on QPPR-generated bounds (LMCI, UMCI), was 1.29 ± 0.27. This ratio was the highest for hexachloroethane (2.39) and the lowest for 1,1-dichloroethylene (1.06).

For the 26 VOCs used in the development of the QPPR, the values of AUC_24_s for a 1 ppm continuous exposure are reported in Table 3. The ratio of the highest to the lowest AUC predicted with *E*_min⁡_ and *E*_max⁡_ was 4.3 ± 1.94 ranging from 1.63 (isoprene) to 8.7 (1,1,2,2-tetrachloroethane). The ratio of the maximum to minimum concentrations predicted using the QPPR metabolism rate was 1.36 ± 0.4 ranging from 1.06 (1,1-dichloroethylene) to 2.8 (isoprene).

**Figure 4:**
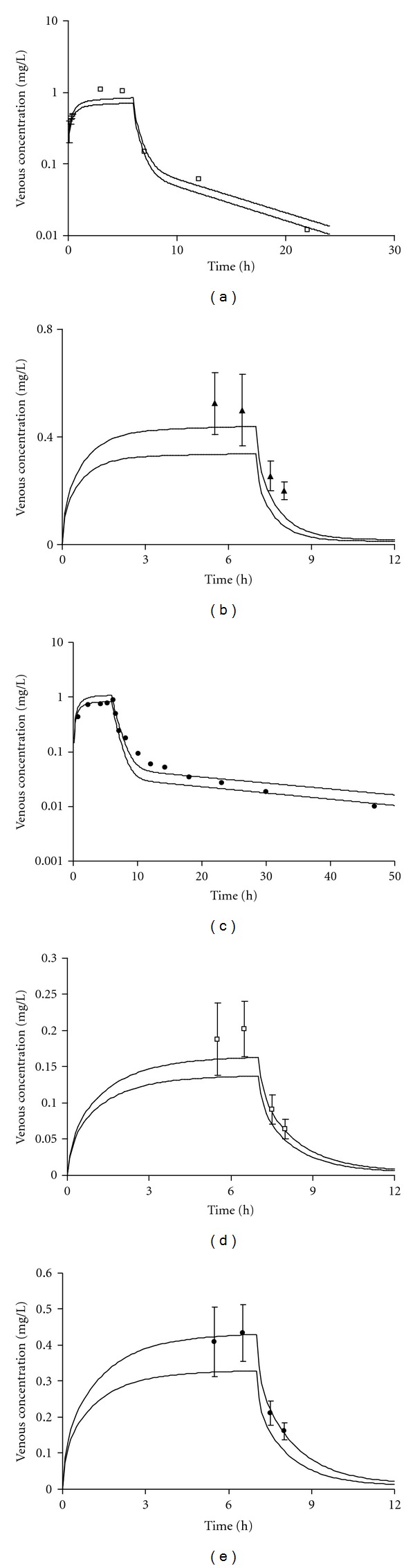
Comparison of PBPK model simulation with experimental data of venous blood concentration following inhalation exposure to (a) 100 ppm, 6 h dichloromethane [77]; (b) 33 ppm, 7 h ethylbenzene [67]; (c) 80 ppm, 6 h styrene [78]; (d) 17 ppm, 7 h toluene [67]; (e) 33 ppm, 7 h *m*-xylene [67]. Bold lines: predicted LMCI and UMCI for *CL*_int⁡_.

Figure 4 illustrates the range of predictions of venous blood pharmacokinetics compared to experimental data [67, 81, 82]. Overall, the predicted envelope of concentrations approximated reasonably the experimental data for dichloromethane, ethylbenzene, styrene, toluene, and *m*-xylene.

### 3.2. Analysis of Applicability of the CL_int⁡_ QPPR to PBPK Modeling

The reliability of applying the QPPR within the PBPK model was assessed for the 26 VOCs in the calibration dataset (Table 4). The uncertainty of the QPPR prediction was estimated as the ratio of predicted *CL*_intPL_ to experimental *CL*_intPL_. For 3 VOCs (isoprene, 1,1-dichloroethylene, and vinyl chloride) the sensitivity of AUC to *CL*_int⁡_ was low (ratio of AUCs < 2) whereas uncertainty of the *CL*_int⁡_ QPPR was low for isoprene and vinyl chloride and medium for 1,1-dichloroethylene. For the other 23 VOCs, the ratio of AUCs was between 2 and 5. For 16 of the later 23 VOCs (benzene; bromochloromethane; bromodichloromethane; chloroform; dibromomethane; 1,2-dichloroethane; hexachloroethane; *n*-hexane; pentachloroethane; styrene; 1,1,1,2-tetrachloroethane; 1,1,2,2-tetrachloroethane; toluene; 1,1,2-trichloroethane; trichloroethylene; *m*-xylene) the prediction uncertainty was low, thus the confidence in using the QPPR in the PBPK model is high for these compounds. The uncertainty was medium for the prediction of *CL*_intPL_ for 7 VOCs (carbon tetrachloride; chloroethane; 1,1-dichloroethane; *cis*-1,2-dichloroethylene; dichloromethane; ethylbenzene; methyl chloride). Therefore, for these chemicals, the confidence in using the QPPR in an inhalation PBPK model to evaluate the AUC is medium. 

### 3.3. QPPR Evaluation

**Figure 5:**
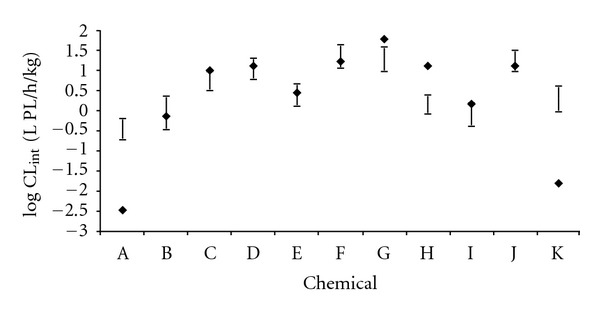
Comparison of the predicted log *CL*_int⁡_ (LMCI and UMCI) with the experimental data on 11 VOCs. The bars represent the QPPR predictions and the symbols the experimental values. A: 1,1,1-trichloroethane; B: 1,2,4-trimethylbenzene; C: 1,2-dichloroethylene (*trans*-); D: bromoform; E: dibromochloromethane; F: ethylene; G: furan; H: halothane; I: *o*-xylene; J: propylene; K: tetrachloroethylene.

The QPPR model was applied to predict the *CL*_intPL_ of 11 VOCs that were not in the calibration dataset.  Table 5 presents the values of the input parameters along with the experimental data for the 11 VOCs used in QPPR evaluation.  Figure 5 illustrates the comparison of the predicted values of *CL*_intPL_ (LMCI and UMCI) and the experimental data. The average difference (± standard deviation) between the UMCI and the LMCI was 0.57 ± 0.11 ranging from 0.46 (bromoform) to 0.84 (1,2,4-trimethylbenzene). The highest UMCI-LMCI ranges were obtained for furan (0.62), tetrachloroethylene (0.63), and 1,2,4-trimethylbenzene (0.84). The nearest predicted values of UMCI and LMCI on log *CL*_intPL_ were greater than 5-fold of the experimental data for tetrachloroethylene (LMCI = 0.02 versus exp⁡ = −1.8). As in the QPPR development section, the impact of the imprecision on these log *CL*_int⁡_ predictions on the pharmacokinetics in humans was evaluated by PBPK modeling. 

**Figure 6:**
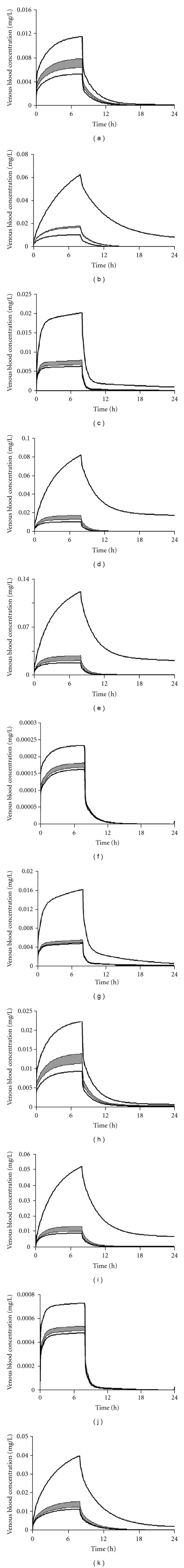
24 h simulation of the venous blood concentration following inhalation exposure to 1 ppm, 8 h for 11 volatile organic compounds considering maximum and minimum (bold lines) and QPPR-based hepatic extraction (grey area). (a) 1,1,1-Trichloroethane; (b) 1,2,4-trimethylbenzene; (c) 1,2-dichloroethylene (*trans*-); (d) bromoform; (e) dibromochloromethane; (f) ethylene; (g) furan; (h) halothane; (i) *o*-xylene; (j) propylene; (k) tetrachloroethylene.

Figure 6 presents the predictions of the 24 h blood pharmacokinetics following 8 h exposure to 1 ppm of each of the 11 VOCs used in the QPPR evaluation. The bold lines represent the simulations obtained using 0 and 1 as the hepatic extraction ratio, whereas the grey area encompassed by thin lines represents the simulation obtained using LMCI and UMCI of predicted *CL*_int⁡_ in PBPK models. The reduction of the region of uncertainty associated with the complete lack of knowledge of hepatic extraction ratio in humans (i.e., ranging from 0 to 1) by the envelope of the concentrations predicted using the QPPR predictions was observed for the 11 VOCs.

The mean ratio (± standard deviation) of the PBPK model simulated values of the end-of-exposure blood concentrations obtained with *E*_min⁡_ and *E*_max⁡_ was 3.92 ± 2.13 ranging from 1.42 (ethylene) to 7.45 (bromoform). However, the same average ratio (± standard deviation) of PBPK simulated blood concentrations, based on QPPR-generated bounds (LMCI and UMCI) was 1.2 ±0.1, ranging from 1.07 (ethylene) to 1.33 (bromoform). 

Table 6 presents the values of the AUC_24_s (mg/L-h) for the 11 VOCs used in the evaluation of the QPPR. The average ratio of the highest to lowest AUC predicted using *E*_min⁡_ and *E*_max⁡_ was 4.08 ± 2.31 (mean ± SD). The lowest and highest ratios, based on the theoretical bounds of hepatic extraction (i.e., *E* = 0.001 or 0.999), were observed for ethylene (1.44) and bromoform (7.96), respectively.

The ratio of the maximum to minimum concentrations predicted using the QPPR metabolism rate was 1.2 ± 0.1, ranging from 1.07 (propylene) to 1.33 (dibromochloromethane).

**Figure 7:**
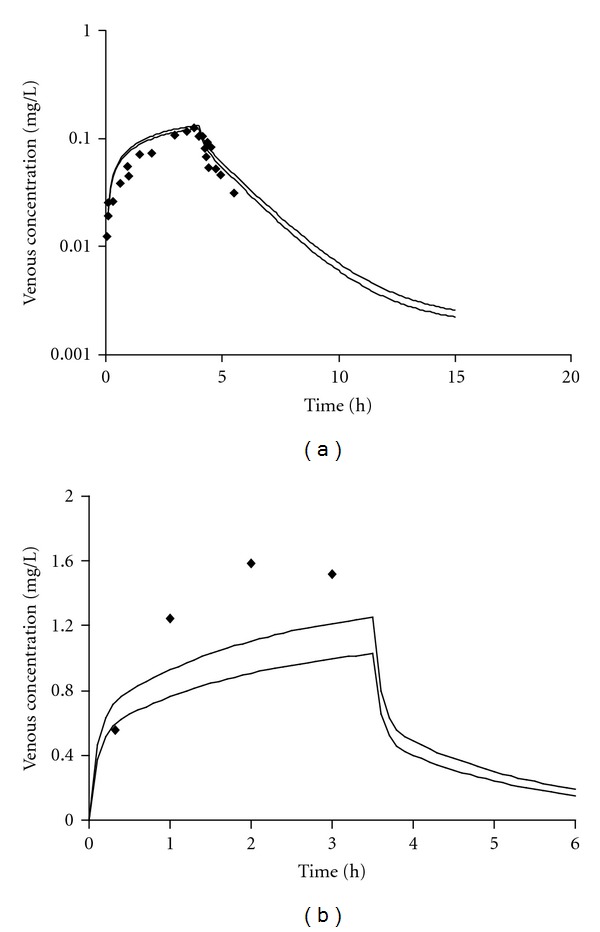
Comparison of PBPK model simulations (bold lines: predicted LMCI and UMCI for *CL*_int⁡_) with experimental data of venous blood concentration following inhalation exposure to (a) 8 ppm, 4 h 1,2,4-trimethylbenzene [59] and (b) 175 ppm, 3.5 h 1,1,1-trichloro ethane [85].

Figure 7 illustrates the range of predictions for two of the chemicals in the external dataset (1,2,4-trimethylbenzene and 1,1,1-trichloroethane) venous blood pharmacokinetics compared to experimental data [61, 85]. The QPPR-PBPK model-generated “envelope” of concentrations simulated reasonably the experimental data for 1,2,4-trimethylbenzene whereas the blood concentrations of 1,1,1-trichloroethane were underestimated by about 30%.

### 3.4. Analysis of Applicability of the *CL*_int⁡_ QPPR to PBPK Modeling

The reliability of applying the QPPR within the PBPK model was assessed for the 11 VOCs in the evaluation dataset, using the framework shown in Figure 1. Considering that the experimental data of *CL*_intPL_ for new or untested chemicals will be essentially unknown, it is realistic to consider the uncertainty of the QPPR prediction of *CL*_intPL_ to be high for all chemicals in the evaluation dataset.

 The results of the analysis of applicability for the chemicals in the evaluation dataset are reported in Table 7. For 3 VOCs (ethylene; propylene; 1,1,1-trichloroethane) the sensitivity was low (ratio of AUCs < 2) thus the reliability of using their *CL*_int⁡_ QPPR in the PBPK was considered high. For the other 8 VOCs (bromoform; dibromochloromethane; *trans*-1,2-dichloroethylene; furan; halothane; tetrachloroethylene; 1,2,4-trimethylbenzene; *o*-xylene), the ratio of the maximum to the minimum possible AUCs was between 2 and 5, such that the confidence in using the QPPR in an inhalation PBPK model to evaluate the AUCs is medium for these chemicals.

## 4. Discussion

SARs, QSAR, QSPRs, and QPPRs have been developed for various toxicological and chemical properties but only very few studies have focused on developing such models to parameterize PBPK models [8, 86]. A limitation in developing PBPK models relates to the availability of the metabolic constants (*CL*_int⁡_, *V*_max⁡_, and *K*_*m*_) [8]. Quantitative relationships between structure and metabolism rates have been investigated for a limited number of closely related compounds, even though their applicability to PBPK modeling has not been demonstrated (e.g., QSPR models for *K*_cat_ and 1/*K*_*m*_ [87]). Other works in this area relate to the development of quantum chemical or quantum dynamic methods for prediction of activation energy or enthalpy of activation of P450 mediated reactions [20, 25, 26, 31, 36, 38, 88–91], which have not been used to derive metabolism constants for direct incorporation within rodent or human PBPK models. 

The use of the group contribution method to develop QSPRs for integration within PBPK models has been successfully demonstrated, particularly for the inhalation toxicokinetics of VOCs [40–43]. This approach however is limited to VOCs containing one or more of the molecular groups or fragments for which the contribution has been evaluated (i.e., CH_3_, CH_2_, CH, C, C=C, benzene ring, H on benzene ring, and halogens). In order to extend the applicability domain then, it is important to investigate the feasibility of developing QSPRs based on more global, physicochemical properties. In this regard, the present study investigated the development of a QPPR, that used chemical properties rather than chemical structure as input, and it was calibrated to predict *CL*_int⁡_ expressed in terms of chemical affinity to phospholipids in the endoplasmic reticulum in which CYP enzymes are embedded [63]. This logical transformation of *CL*_int⁡_ data, reported here for the first time in literature, facilitated the development of more adequate QPPR than the conventional *CL*_int⁡_ based on blood concentrations. All efforts to develop QPPRs for predicting *CL*_int⁡_ based on blood concentrations were unsuccessful. The QPPR based chemical affinity to phospholipids–obtained in this study should be regarded as a screening level tool to provide plausible range of metabolism rates in order to facilitate a first-cut evaluation of the blood concentration of inhaled VOCs in humans. The uncertainty associated with this QPPR tool should be evaluated along with the sensitivity of *CL*_int⁡_ on the dose metrics of the chemical of interest, in the perspective of intended precision. Accordingly, if the dose metric is highly sensitive to *CL*_int⁡_ and the QPPR predictions of *CL*_int⁡_ are highly uncertain, then the present tool is of limited use even for screening purposes. In such cases, then *in vivo* or *in vitro* studies can be undertaken to get chemical-specific estimates of *CL*_int⁡_.

The QPPR predictions were reasonably in accordance with experimental values for most but not all chemicals in the calibration and evaluation datasets. For some chemicals, the predicted values of log *CL*_int⁡_ for 1,1,1-trichloroethane (Figure 5(A)) and tetrachloroethylene (Figure 5(K)) exceeded the experimental values by two orders of magnitude. The QSPR for rat hepatic clearance developed by Béliveau and colleagues [41] also overestimated the metabolic rate of these two VOCs. However the PBPK model for 1,1,1-trichlorethane indicated that the AUC of parent chemical in venous blood is not sensitive to *V*_max⁡_ and *K*_*m*_ [92, 93]. This was demonstrated in Figure 6(a), showing that QPPR-overestimation of *CL*_intPL_ of 1,1,1-trichloroethane led only to a minimal impact, in terms of the underestimation of the venous blood concentration. In the case of tetrachloroethylene, a poorly metabolized halogenated VOC, the overestimation of the *CL*_intPL_ led to a 3-fold underestimation of the *C*_max⁡_ (Figure 6(k)) or a 4-5-fold underestimation of the AUC_24_ (Table 7). If this magnitude of error is not acceptable for screening-level evaluation, then the metabolic rate should be experimentally determined. The combined assessment of the uncertainty/sensitivity of metabolic constants in PBPK models would facilitate the determination of the applicability of the QPPR model, given the level of precision need for an application (Figure 1)

The QPPR developed in this study is a generic tool to provide initial estimates of *CL*_int⁡_ of VOCs metabolized by hepatic CYP. It does not take into account stereochemistry or other pathway-specific rates and processes, which may be important for some chemicals (e.g., predicted values of *CL*_int⁡_ are almost identical for 1,1-dichloroethylene and *cis*-1,2-dichloroethylene but experimental values vary by log units of 1.06). Therefore, predictions of *CL*_int⁡_ based on generic considerations are likely to be inaccurate for specific chemicals but are of limited use in that the estimates (along with the bounds, representing the level of uncertainty) can be integrated with human physiology to provide a first-cut view of the plausible kinetic profiles. 

The utility of the QPPR models depends, in part, on the ability to reproducibly calculate the descriptors [74]. Hence, in this study, the descriptors that could be easily calculated and interpreted were chosen and obtained using EPISUITE (for log *P*_ow  _and *P*_wa_) and MMPro (for the ionization potential). However, the blood solubility parameter (i.e., blood : air PC) is additionally required and this can either be obtained experimentally *in vitro* or using other QSARs that account for protein (i.e., haemoglobin and plasma protein) binding in addition to solubility considerations. There are some algorithms and QSARs available in this regard, but further development is necessary to adequately account for the protein binding phenomena in human blood for various classes of chemicals [8].

The QPPR developed in this study computes *CL*_intPL_, which can then be converted to *CL*_intblood_ for use in PBPK modeling. In an effort to evaluate whether the same input parameters can be used to relate to *CL*_intblood_, additional analyses were performed. These yielded the following equation (significant terms only):

(6) $$\log\;{CL}_{\text{intblood}}=5.117-0.305\cdot \log P_{\text{ow}}-0.324\cdot \text{IP}.$$

Even though (5) and (6) give almost identical results (one for *CL*_intPL_ and the other for *CL*_intblood_) despite the differing *R* ^2^ values (0.796 versus 0.402), it should be noted that (5) was obtained based on statistical analysis of calibration dataset (i.e., modeling) whereas (6) was derived simply by fitting *CL*_intblood_ to the specific input parameters. Further rearrangements and simplifications of the QPPR, as well as the loss of accuracy associated with such attempts, were not performed in the current study. 

The output of the QPPR developed in the present study is log *CL*_int⁡_, which is useful for simulating pharmacokinetics in humans of chemicals at low levels of exposure. *CL*_int⁡_ is applicable to first-order situations (i.e., when blood levels in humans are much lower than the *K*_*m*_ for metabolizing enzyme) and is derived by dividing the *V*_max⁡_ (i.e., the enzyme turn-over) with *K*_*m*_ (representing the affinity of the substrate for the enzyme). The input parameters of the QPPR, namely, log *P*_ow_ and log *P*_bw_, are estimates of the relative solubility in octanol, water, and blood. Then, an interpretation of the model for *CL*_int⁡_ could be that the binding to the P450 enzyme is a result of hydrophobic interactions [94] which, in turn, can be estimated with parameters reflective of the solubility in *n*-octanol and blood. The solubility in blood is the sum of the solubility in its components (water, phospholipids, neutral lipids, and proteins). Most of the studied VOCs are likely to bind to hemoglobin because of their lipophilicity (log *P*_ow_ value above 1) and low molecular volume [40]. The *P*_bw_, thus, is likely an indicator of the binding to proteins, whereas the log *P*_ow_ reflects more the affinity for biotic lipids in the metabolism microenvironment. Similar to log *P*_ow_, the ionization potential has already been correlated with metabolic rates, namely, the *V*_max⁡_ and *V*_max⁡_/*k*_*m*_ [23], as this latter parameter could be correlated with the energy needed to break a covalent bond for the oxidation of the substrate.

The QSPR model for *CL*_int⁡_ developed in this study has a defined theoretical endpoint, is nonambiguous, has a defined domain of application, was analyzed using appropriate goodness-of-fit (*R* ^2^) and robustness (*Q* ^2^), and has an attempt of mechanistic interpretation. The *in vivo* dataset on 26 VOCs used for the QPPR calibration was chosen because it was previously collated and used in QSPR analyses [40, 41]. These values were taken mainly from the work of Gargas et al. [24]. The QSPR analysis was also attempted with the entire dataset of 37 VOCs (calibration + external dataset) but it did not improve the goodness-of-fit statistics (not shown).

The predicted bounds of the 95% confidence interval of intrinsic clearance were incorporated within a PBPK model to predict the blood toxicokinetics of VOCs. The simulations of blood kinetics were comparable to experimental data for 6 VOCs (toluene, *m*-xylene, ethylbenzene, styrene, dichloromethane,1,2,4-trimethylbenzene, and 1,1,1-trichloroethane, Figures 4 and 7). The simulations obtained in the present study, using lower and upper confidence intervals on the mean predicted *CL*_int⁡_, reduced clearly the uncertainty bounds associated with the total lack of knowledge (i.e., *E* ranging anywhere between 0 and 1). Furthermore, the present study incorporated the QPPR predictions of *CL*_int⁡_ along with physiological parameters, such that impact on *in vivo* kinetics could be simulated. In effect, in some cases where the uncertainty on *CL*_int⁡_ predictions was high, it did not translate into a proportionate error on the predictions of kinetics, due to the additional consideration of physiological constraints, and such observations are critical in data-poor situations for designing focused studies to generate chemical-specific data *in vitro* or *in vivo*. 

The QPPR developed in this study approximated the experimental rat metabolic constants for the various low-molecular-weight VOCs; and it was used along with the human physiology to generate initial or screening level values of *CL*_int⁡_ to construct human PBPK models that could be of potential use to interpret data such as measured biomarker levels or for designing kinetic studies to reduce database uncertainty. As shown with some VOCs (e.g., Figure 3: 1,1,1,2-tetrachloroethane, hexachloroethane, and *n*-hexane), the blood concentration profile is extremely influenced by *CL*_int⁡_, such that metabolism cannot be neglected in simulating or interpreting human exposure data. And in such cases, the ability to generate at least a range of plausible values of *CL*_int⁡_, as done in the present study, would facilitate first in-human simulations of pharmacokinetics of parent chemicals. Integrating information on the impact of metabolism on dose metrics (i.e., AUC) along with prediction uncertainty of the QPPR facilitates the determination of the level of confidence in using this screening level tool. Depending upon the overall confidence in the QPPR application for predicting dose metrics (low, medium, and high) relative to the use purposes, decisions can be made as to the specific studies needed. 

Overall, the QPPR developed in the present study allows to predict the *CL*_int⁡_ of VOCs on the basis of generic molecular descriptors rather than with fragment constants as done previously. The chemical concentration in phospholipids, for the first time, was found to be a dose metric amenable to QPPR analysis. The QPPR was then used to generate range of values of *CL*_int⁡_; the level of confidence in these estimates was assessed by considering the impact of *CL*_int⁡_ on the simulated dose metrics (i.e., AUC of parent chemical in venous blood). For other dose metrics and situations, a more robust QPPR needs to be developed, and such efforts can be based on the methodological developments accomplished in this study. The QPPR-based simulation of pharmacokinetics reduced the range of uncertainty for few substances relative to complete lack of knowledge of the *CL*_int⁡_, but it needs to be evaluated/refined with much larger dataset should this screening-level approach be adopted for providing more precise estimates of metabolism rates. Overall, the integrated QPPR-PBPK model developed in this study is a potentially useful tool for characterizing and reducing the uncertainty associated with the complete lack of knowledge of *CL*_int⁡_ in predicting human pharmacokinetics of inhaled VOCs.

## Supplementary Material

The code of the human PBPK model for inhaled VOCs is provided in this supplemental material. The model is written in advance continuous simulation language to be run in ACSL^®^ (acslX^®^, version 2.5, Aegis Technologies Group, Inc, Huntsville, AL). The model parameters are defined in the “initial” section and the differential equations are written in the “dynamic” section. PBPK consisted in four tissue compartments (liver, fat, richly, and poorly perfused tissues) and a gas exchange lung interconnected by blood flows. Two descriptions of hepatic clearance are available: one using the intrinsic clearance (CLint) and the other the extraction ratio.

## Figures and Tables

**Figure 3:**

24 h simulation of the venous blood concentration following inhalation exposure to 1 ppm, 8 h for 26 volatile organic compounds considering maximum and minimum (bold lines) and QPPR-based hepatic extraction (grey area). (a) 1,1,1,2-Tetrachloroethane; (b) 1,1,2,2-tetrachloroethane; (c) 1,1,2-trichloroethane; (d) 1,1-dichloroethane; (e) 1,1-dichloroethylene; (f) 1,2-dichloroethane; (g) 1,2-dichloroethylene (*cis*-); (h) benzene; (i) bromochloromethane; (j) bromodichloromethane; (k) carbon tetrachloride; (l) chloroethane; (m) chloroform; (n) dibromomethane; (o) dichloromethane; (p) ethylbenzene; (q) hexachloroethane; (r) isoprene; (s) methyl chloride; (t) *m*-xylene; (u) *n*-hexane; (v) pentachloroethane; (w) styrene; (x) toluene; (y) trichloroethylene; (z) vinyl chloride.

**Table 1:** Partition coefficients used in the human PBPK models.

Chemicals^a^	Partition coefficient (PC)^b^						Reference
	*P*_ba_	*P*_lb_	*P*_rb_	*P*_pb_	*P*_fb_	*P*_plb_	
Benzene	8.19	2.08	2.08	1.26	60.93	4.55	[66]
Bromochloromethane	10.4	2.81	2.81	1.07	31.5	2	[66, 77]
Bromodichloromethane	26.6	1.15	1.15	0.47	19.77	2.48	[78, 79]
Carbon tetrachloride	2.73	5.2	5.2	1.67	131.5	8.57	[66]
Chloroethane	2.69	1.34	1.34	1.2	14.3	4.42	[66]
Chloroform	6.85	3.08	3.08	2.03	29.6	1.93	[66]
Dibromomethane	19.9	3.42	3.42	2.03	39.8	1.78	[66, 77]
Dichloroethane (1,1-)	4.94	2.19	2.19	1.04	33.2	4.27	[66]
Dichloroethane (1,2-)	19.5	1.83	1.83	1.2	17.64	9.25	[66]
Dichloroethylene (1,1-)	0.81	5.46	5.46	2.53	84.69	4.82	[66, 77]
Dichloroethylene (*cis*-1,2)	9.85	1.55	1.55	0.62	23	5.2	[66]
Dichloromethane	9.7	1.46	1.46	0.82	12.4	1.79	[80]
Ethylbenzene	28	2.99	2.15	0.93	55.6	13.2	[67]
Hexachloroethane	52.4	7.04	7.04	1.43	63.4	156	[66]
Hexane (*n*-)	2.13	5.2	5.2	2.9	159	1.89	[47]
Isoprene	0.75	2.57	2.45	1.97	82	11.84	[52]
Methyl chloride	2.48	1.4	1.4	0.39	5.44	3.04	[66]
Pentachloroethane	50.3	5.17	5.17	1.44	81.9	21.7	[66, 77]
Styrene	52	2.7	5.7	1	50	30.2	[50]
Tetrachloroethane (1,1,1,2-)	30.2	2.92	2.92	1.31	71.1	37.3	[66]
Tetrachloroethane (1,1,2,2-)	116	1.69	1.69	0.87	32.47	14.3	[66]
Toluene	15.6	5.36	5.36	1.77	65.4	13.8	[67]
Trichloroethane (1,1,2-)	35.7	2.05	2.05	0.64	40.3	10.1	[66]
Trichloroethylene	8.11	3.35	3.35	1.24	68.3	5.73	[66]
Vinyl chloride	1.16	1.38	1.38	1.81	17.2	4.87	[66]
Xylene (*m*-)	26.4	3.44	3.44	1.59	70.4	15.1	[67]
*Bromoform*	102.3	2.06	2.06	1.12	40.4	2.44	[78, 79]
*Dibromochloromethane*	49.2	2.56	2.56	1.13	38.96	1.48	[78, 79]
*Dichloroethylene (trans-1,2-)*	6.04	1.48	1.48	0.58	24.5	11.7	[66]
*Ethylene*	0.22	2.05	2.18	2.95	8.73	1.05	[59]
*Furan*	6.59	0.9	0.9	0.64	9.72	2.75	[56]
*Halothane*	3.3	2.42	2.42	2.91	44.2	5.82	[58]
*Propylene*	0.44	1.09	1.2	1.25	11.7	1.52	[54]
*Tetrachloroethylene*	10.3	5.88	5.88	3.1	119.1	11.1	[57]
*Trichloroethane (1,1,1-)*	2.53	1.24	3.4	3.4	103.9	21.7	[48]
*Trimethylbenzene (1,2,4-)*	85	4.4	4.4	2.11	109	19	[61]
*Xylene (*o*-) *	34.9	3.09	3.09	1.47	53.8	22.6	[66]

^a^ Chemicals in italics were not included in the dataset for the calibration of the model.

^b^ *P*_ba_: blood : air PC; *P*_lb_: liver blood PC; *P*_rb_   : richly perfused tissues : blood PC; *P*_pb_: poorly perfused tissues : blood PC; *P*_fb_: fat : blood PC; *P*_plb_: phospholipids : blood PC.

**Table 2:** Input parameters and experimental data of log *CL*_*intPL*_.

Chemical	Input parameters			Log *Cl*_intPL_^a^ (L_PL_/h/kg)	Ref *V*_max⁡_, *K*_*m*_
	Log *P*_ow_	Log *P*_bw_	Ionization potential (eV)		
Benzene	1.99	0.820	9.743	0.667	[53]
Bromochloromethane	1.43	0.642	10.562	1.118	[49]
Bromodichloromethane	1.61	0.717	10.676	1.029	[46]
Carbon tetrachloride	2.44	0.988	10.985	−0.700	[24]
Chloroethane	1.58	0.438	10.410	0.987	[24]
Chloroform	1.52	0.741	10.839	1.192	[24]
Dibromomethane	1.52	0.777	10.587	1.275	[51]
Dichloroethane (1,1-)	1.76	0.624	10.577	0.974	[24]
Dichloroethane (1,2-)	1.83	0.356	10.446	0.163	[24]
Dichloroethylene (1,1-)	2.12	0.922	9.748	1.223	[24]
Dichloroethylene (*cis*-1,2)	1.98	0.752	9.493	0.092	[24]
Dichloromethane	1.34	0.608	10.582	0.777	[24]
Ethylbenzene	3.03	1.386	9.406	−0.334	[49]
Hexachloroethane	4.03	1.315	10.843	−1.767	[24]
Hexane (*n*-)	3.29	2.492	11.276	0.252	[47]
Isoprene	2.58	0.987	9.349	0.472	[52]
Methyl chloride	1.09	0.160	10.473	0.299	[24]
Pentachloroethane	3.11	1.251	10.763	−0.297	[24]
Styrene	2.89	0.889	9.130	−0.088	[50]
Tetrachloroethane (1,1,1,2-)	2.93	0.836	10.728	−0.693	[24]
Tetrachloroethane (1,1,2,2-)	2.19	0.519	10.736	0.051	[24]
Toluene	2.54	0.879	9.442	0.282	[49]
Trichloroethane (1,1,2-)	2.01	0.491	10.689	0.018	[24]
Trichloroethylene	2.47	1.192	9.368	0.916	[24]
Vinyl chloride	1.62	0.433	9.833	0.741	[24]
Xylene (*m*-)	3.09	1.388	9.308	0.218	[48]

^a^: EXP. experimental data (references in Section 2); LMCI and UMCI: lower and upper bound of the 95% mean confidence interval, respectively.

**Table 3:** Area under the curve for four metabolic scenarios for the VOCs used in the QPPR development.

Chemicals	24 h Area under the curve (mg/L-h)			
	Metabolic scenario^a^			
	*E*_min⁡_	*E*_max⁡_	LMCI	UMCI
Benzene	0.437	0.125	0.155	0.138
Bromochloromethane	0.913	0.229	0.305	0.257
Bromodichloromethane	2.380	0.356	0.523	0.422
Carbon tetrachloride	0.34	0.153	0.224	0.186
Chloroethane	0.156	0.069	0.084	0.076
Chloroform	0.599	0.185	0.257	0.211
Dibromomethane	1.659	0.336	0.466	0.384
Dichloroethane (1,1-)	0.391	0.138	0.181	0.158
Dichloroethane (1,2-)	1.142	0.205	0.305	0.241
Dichloroethylene (1,1-)	6.98 × 10^−2^	4.22 × 10^−2^	4.76 × 10^−2^	4.47 × 10^−2^
Dichloroethylene (*cis*-1,2)	0.702	0.174	0.214	0.188
Dichloromethane	0.65	0.157	0.206	0.174
Ethylbenzene	1.247	0.216	0.32	0.246
Hexachloroethane	3.071	0.494	1.774	0.738
Hexane (*n*-)	0.15	0.073	0.129	0.084
Isoprene	4.59 × 10^−2^	2.81 × 10^−2^	0.084	2.95 × 10^−2^
Methyl chloride	0.119	0.054	0.064	0.057
Pentachloroethane	2.584	0.418	0.929	0.595
Styrene	1.497	0.222	0.322	0.246
Tetrachloroethane (1,1,1,2-)	1.911	0.337	0.739	0.457
Tetrachloroethane (1,1,2,2-)	3.337	0.384	0.717	0.495
Toluene	0.74	0.168	0.223	0.188
Trichloroethane (1,1,2-)	1.876	0.284	0.474	0.355
Trichloroethylene	0.721	0.209	0.267	0.227
Vinyl chloride	6.68 × 10^−2^	3.79 × 10^−2^	4.25 × 10^−2^	3.98 × 10^−2^
Xylene (*m*-)	1.117	0.209	0.308	0.236

^a^: *E*_min⁡_: no metabolism; *E*_max⁡_: maximum hepatic extraction; LMCI and UMCI: lower and upper bound of the 95% mean confidence interval, respectively.

**Table 4:** Reliability analysis of the QPPR for *CL*_*int*_ on the PBPK predicted AUC.

		Impact of metabolism on AUC (AUC_*E*_min⁡__/AUC_*E*_max⁡__)^a^		
		Low (<2)	Medium (2–5)	High (>5)
QPPR prediction uncertainty (Pred./Exp. CL*_intPL_*)^b^	Low (<2)	Isoprene, vinyl chloride	Benzene; bromochloromethane; bromodichloromethane; chloroform; dibromomethane; 1,2-dichloroethane; hexachloroethane; *n*-hexane; pentachloroethane; styrene; 1,1,1,2-tetrachloroethane; 1,1,2,2-tetrachloroethane; toluene; 1,1,2-trichloroethane; trichloroethylene; *m*-xylene	
	Medium (2–5)	1,1,-Dichloroethylene	Carbon tetrachloride; chloroethane; 1,1-dichloroethane; *cis*-1,2-dichloroethylene; dichloromethane; ethylbenzene; methyl chloride	
	High (>5)			

^a^ Calculated as the ratio of the PBPK simulations of 24 h AUC (of venous blood concentration, 1 ppm VOC, 24 h exposure) obtained by setting *E* = 0 (i.e., *CL*_int⁡_ = 0) to that setting *E* = 1 (i.e., *CL*_int⁡_ = 1000).

^b^ Calculated as the ratio of the predicted to the experimental values of *CL*_intPL_.

**Table 5:** Input parameters and experimental data on log *CL*_*int*_ for VOCs of QPPR evaluation.

Chemical	Input parameters			Log *Cl*_intPL_^a^ (L_PL_/h/kg)	Ref *V*_max⁡_, *K*_*m*_
	log *P*_ow_	log *P*_bw_	IP (eV)		
Bromoform	1.79	0.896	10.837	1.006	[46]
Dibromochloromethane	1.70	1.025	10.702	1.108	[46]
Dichloroethylene (*trans*-1,2-)	1.98	0.484	9.512	0.438	[55]
Ethylene	1.27	0.776	10.638	1.208	[59]
Furan	1.36	0.441	9.375	1.773	[56]
Halothane	2.26	0.977	11.039	1.104	[58]
Propylene	1.68	0.995	10.103	1.118	[54]
Tetrachloroethylene	2.97	1.404	9.217	−1.804	[57]
Trichloroethane (1,1,1-)	2.68	0.823	10.751	−2.467	[48]
Trimethylbenzene (1,2,4-)	3.63	1.829	9.084	−0.132	[61]
Xylene (*o*-)	3.09	1.213	9.304	0.163	[60]

^a^ Experimental data (references in Section 2).

**Table 6:** Area under the curve for four metabolic scenarios, for VOCs in the evaluation dataset.

	24 h Area under the curve (mg/L-h)			
Chemicals	Metabolic scenario^a^			
	*E*_min⁡_	*E*_max⁡_	LMCI	UMCI
Bromoform	2.122	0.267	0.434	0.328
Dibromochloromethane	3.114	0.45	0.7	0.527
Dichloroethylene (*trans*-1,2-)	0.472	0.149	0.185	0.16
Ethylene	5.87 × 10^−3^	4.08 × 10^−3^	4.55 × 10^−3^	4.23 × 10^−3^
Furan	0.388	0.113	0.131	0.118
Halothane	0.523	0.22	0.325	0.266
Propylene	1.76 × 10^−2^	1.16 × 10^−2^	1.3 × 10^−2^	1.21 × 10^−2^
Tetrachloroethylene	0.953	0.266	0.363	0.291
Trichloroethane (1,1,1-)	0.271	0.125	0.184	0.15
Trimethylbenzene (1,2,4-)	1.577	0.249	0.425	0.395
Xylene (*o*-)	1.297	0.218	0.325	0.248

^a^: *E*_min⁡_: no metabolism; *E*_max⁡_: maximum hepatic extraction; LMCI and UMCI: lower and upper bound of the 95% mean confidence interval, respectively.

**Table 7:** Reliability analysis for the chemicals in the QPPR evaluation dataset.

		Impact of metabolism on AUC (AUC_*E*_min⁡__/AUC_*E*_max⁡__)^a^		
		Low (<2)	Medium (2–5)	High (>5)
	Low (<2)			
QPPR prediction uncertainty (Pred./Exp. *CL*_intPL_)^b^	Medium (2–5)			
	High (>5)	Ethylene; propylene; 1,1,1-trichloroethane	Bromoform; dibromochloromethane; *trans*-1,2-dichloroethylene; furan; halothane; tetrachloroethylene; 1,2,4-trimethylbenzene; *o*-xylene	

^a^ Calculated as the ratio of the PBPK simulations of 24 h AUC (of venous blood concentration, 1 ppm VOC, 24 h exposure) obtained by setting *E* = 0 (i.e., *CL*_int⁡_ = 0) to that setting *E* = 1 (i.e., *CL*_int⁡_ = 1000).

^b^ Calculated as the ratio of the predicted to the experimental values of *CL*_intPL_.
