# Supplementary material for: Quantitative Property-Property Relationship for Screening-Level Prediction of Intrinsic Clearance of Volatile Organic Chemicals in Rats and Its Integration within PBPK Models to Predict Inhalation Pharmacokinetics in Humans
Source: J Toxicol. 2012 May 22;2012:286079. doi: 10.1155/2012/286079 (PMC3364689; doi:10.1155/2012/286079)
Supplement: Supplementary file 1 — The code of the human PBPK model for inhaled VOCs is provided in this supplemental material. The model is written in advance continuous simulation language to be run in ACSL® (acslX®, version 2.5, Aegis Technologies Group, Inc, Huntsville, AL). The model parameters are defined in the “initial” section and the differential equations are written in the “dynamic” section. PBPK consisted in four tissue compartments (liver, fat, richly, and poorly perfused tissues) and a gas exchange lung interconnected by blood flows. Two descriptions of hepatic clearance are available: one using the intrinsic clearance (CLint) and the other the extraction ratio. [file 286079.f1.pdf]

SUPPLEMENTARY DATA

INITIAL

```
constant BW = 70.            ! Body weight (kg)
constant conc = 0            ! Exposition dose (ppm)
constant MW = 0              ! Molecular weight
constant SV = 24.450         ! Standard volume mL/mol
constant DUREE= 0            ! Exposure lenght (h)
constant periode = 0         ! Period between two exposures (h)
```

! Tissue volumes (fraction of body weight)

```
constant VLc = 0.026         ! Liver (fraction of body weight)
constant VRc = 0.05          ! Richly perfused tissues (fraction of body weight)
constant VSc = 0.62          ! Poorly perfused tissues (fraction of body weight)
constant VFc = 0.19          ! Fat (fraction of body weight)

VL = VLc*BW                  ! Liver (L)
VR = VRc*BW                  ! Richly perfused tissues (L)
VS = VSc*BW                  ! Poorly perfused tissues (L)
VF = VFc*BW                  ! Fat (L)
```

! Flows

constant QPc = 18 ! Alveloar ventilation (L/h/kg)

constant QCc = 18 ! Cardiac output (L/h/kg)

QP = QPc*BW**0.74 ! Alveloar ventilation (L/h)

QC = QCc*BW**0.74 ! Cardiac output (L/h)

constant QLc = 0.26 ! Liver (fraction of QC)

constant QRc = 0.44 ! Richly perfused tissues (fraction of QC)

constant QSc = 0.25 ! Poorly perfused tissues (fraction of QC)

constant QFc = 0.05 ! fat (%QC)

QL = QLc*QC! Liver (L/h)

QR = QRc*QC ! Richly perfused tissues (L/h)

QS = QSc*QC ! Poorly perfused tissues (L/h)

QF = QFc*QC ! fat (L/h)

! Partition coefficients (PCs)

constant PB = 0.0 ! Blood:air

constant PLB = 0.0 ! Liver:blood

constant PRB = 0.0 ! RPT:blood

constant PSB = 0.0 ! PPT:blood

constant PFB = 0.0 ! Fat:blood

! Metabolism constants

constant logclint = 0.0 ! log CLint (L PL/h/kg)

CLintPL = 10**logclint ! CLint L PL/h/kg

constant pplb = 0.0 ! Phospholipids:blood PC

CLint = CLintPL * pplb * BW**0.75 ! CLint (L bloob/h)

constant KFC = 0.0 ! First order metabolism (h-kg)-1

KF = KFC * BW**0.3 ! First order metabolism (h-1)

! Hepatic clearance based on CLint and Kf

CLH = QL * (CLINT + KF * VL)/(QL + CLINT + KF * VL)

! Hepatic clearance based extraction ratio (E)

constant E = 0.0 ! Extraction ratio

! CLH = QL * E

END ! INITIAL

DYNAMIC

! Exposure scenario

JOURNE=PULSE(0.,periode,DUREE) ! 24 h exposure

CI=JOURNE*conc *MW / SV /1000 ! Inhaled concentration (mg/L)

! Blood concentrations

CA = (CI*QP+QC*CV)/(QC+QP/PB) ! Arterial mg/L

CV = (QL*CVL+QR*CVR+QS*CVS+QF*CVF)/QC ! Venous mg/L

AUC = INTEG(CV, 0.0) ! Venous blood area under the curve (mg/L-h)

CALV = CA/PB*SV/MW*1000 ! Alveolar air PPM

! Tissue compartments

! Liver

rAL = QL*(CA-CVL) - CLH * CA ! Rate of amount (mg/h)

AL = integ (rAL,0.0) ! Amount (mg)

CL = AL/VL ! Liver concentration (mg/L)

CVL = CL/PLB ! Liver venous blood concentration (mg/L)

! Richly perfused tissues

```
rAR = QR*(CA-CVR)        ! Rate of amount (mg/h)
AR = integ (rAR, 0.0)    ! Amount (mg)
CR = AR/VR               ! Richly perfused tissues concentration (mg/L)
CVR = CR/PRB     ! Richly perfused tissues venous blood concentration (mg/L)
```

! Slowly perfused tissues

```
rAS = QS*(CA-CVS)        ! Rate of amount (mg/h)
AS = integ (rAS, 0.0)    ! Amount (mg)
CS = AS/VS               ! Poorly perfused tissues concentration (mg/L)
CVS = CS/PSB     ! Poorly perfused tissues venous blood concentration (mg/L)
```

! Fat

```
rAF = QF*(CA-CVF)        ! Rate of amount (mg/h)
AF = integ (rAF, 0.0)    ! Amount (mg)
CF = AF/VF               ! Fat concentration (mg/L)
CVF = CF/PFB             ! Fat venous blood concentration (mg/L)
```

```
END ! DYNAMIC
```
